# Sensory Impairment and Algorithmic Classification of Early Cognitive Impairment in Middle-Aged and Older Adults

**DOI:** 10.1093/geroni/igab046.1697

**Published:** 2021-12-17

**Authors:** Yurun Cai, Yuri Agrawal, Jennifer Schrack, Alden Gross, Nicole Armstrong, Eleanor Simonsick, Susan Resnick

**Affiliations:** 1 Johns Hopkins Bloomberg School of Public Health, Baltimore, Maryland, United States; 2 Otolaryngology, Baltimore, Maryland, United States; 3 Warren Alpert Medical School of Brown University, Providence, Rhode Island, United States; 4 National Instute on Aging/NIH, Baltimore, Maryland, United States; 5 National Institute on Aging, Baltimore, Maryland, United States

## Abstract

Sensory function has been linked to cognitive impairment and dementia, but the link between multiple sensory impairments and early cognitive impairment (ECI) is unclear. Sensory function (vision, hearing, vestibular, proprioception, and olfaction) was measured in 390 BLSA participants (age=75±8 years; 57% women; 69% white) from 2012 to 2018 over a mean 3.6 years. ECI was defined based on 1 standard deviation below age-and race-specific means in Card Rotations or California Verbal Learning Test immediate recall. Cox proportional hazard models examined the risk of ECI for each sensory impairment and across categories of impairments. Vision impairment (vs. no vision impairment) was associated with a 70% greater risk of ECI (HR=1.70, p=0.05). Participants with 1 or ≥2 sensory impairments had triple the risk of ECI (HR=3.74 and 3.44, p=0.008 and 0.02, respectively) compared to those without impairment. Future studies are needed to examine whether treatment for sensory impairments can modify these risks.

